# Abdominal ectopic pregnancy following a frozen embryo transfer cycle: a case report

**DOI:** 10.1186/s12884-021-04133-5

**Published:** 2021-10-22

**Authors:** Yan Li, Jiaxuan Geng, Qiaohua He, Jin Lu, Jin Xu, Ying Zhang, Cuilian Zhang

**Affiliations:** Reproductive Medical Center, People’s Hospital of Zhengzhou University, Henan Provincial People’s Hospital, Weiwu Road, Zhengzhou, 450003 Henan China

**Keywords:** Abdominal ectopic pregnancy, Human chorionic gonadotropin, Frozen embryo transfer, Artificial cycle, Case report

## Abstract

**Background:**

Abdominal ectopic pregnancy (AEP) is a rare form of ectopic pregnancy. As the number of in-vitro fertilization (IVF) procedures continues to increase, the incidence of AEP will also rise. However, the rarity and atypical presentation of AEP make early diagnosis challenging.

**Case presentation:**

Herein, we report an AEP following frozen-thawed embryo transfer (FET) in an artificial cycle. The patient was misdiagnosed with implantation failure when the serum human chorionic gonadotropin (hCG) level was detected as 2.59mIU/ml at fourteenth day after embryo transfer. Therefore, she was suggested to stop luteal phase support. However, a ruptured AEP was developed 33 days following embryo transfer, which was diagnosed by laparoscopic surgery.

**Conclusions:**

The case highlighted the delayed serum β-hCG and massive intraperitoneal hemorrhage may be clues to make early diagnosis of AEP. Clinicians must attach great importance to close monitoring and bear in mind the possibility of abdominal pregnancy.

## Background

Abdominal ectopic pregnancy is a rare type of ectopic pregnancy with a reported incidence of 1:10,000 to 1:30,000 pregnancies, and accounts for approximately 1% of ectopic pregnancies [1]. The rarity and atypical presentation of AEP make diagnosis and treatment challenging [2, 3]. As the number of IVF procedures continues to increase, the incidence of AEP will also rise [3]. It is extremely important to make an early diagnosis because of the associated high maternal mortality rate [4]. AEP is classified as either primary or secondary. A primary abdominal pregnancy must meet the following criteria defined by Studdiford in 1942 (1) the presence of normal tubes and ovaries, (2) no evidence of an uteroperitoneal fistula, and (3) the presence of a pregnancy related exclusively to the peritoneal surface and early enough in gestation to eliminate the possibility of secondary implantation after the primary nidation of the tube [5]. However, there are still relatively few reports of abdominal ectopic pregnancies. Herein, we report a case of ruptured primary AEP at thirty-third day after frozen-thawed day-3 embryo-transfer in an artificial cycle with an extremely low serum β-hCG level of 2.59mIU/ml detected at fourteenth day following embryo transfer. The aim of this research is to highlight the possible delayed rise in serum β-hCG in AEP, and summarized our diagnostic experience of this rare type of ectopic pregnancy in early gestational stage.

## Case presentation

A 26-year old woman with primary infertility for 3 years was referred to our reproductive medical center for IVF treatment in 2020. She had spontaneous menses every 30–35 days. The body mass index was 21 kg/m^2^. The transvaginal ultrasound (TVUS) showed bilateral antral follicle count was 13 and a left ovarian endometrial cyst with diameter of approximately 2 cm. The husband’s semen analysis was normal. In her past medical history, she had a surgical history of appendectomy in 2014. She underwent hysterosalpingography (HSG) in 2018 and left hydrosalpinx was suspected on HSG. Subsequently, hysterolaparoscopy was performed in 2019 to exclude tubal pathology. Normally shaped uterus and patent oviducts were found during laparoscopy surgery. During the consultation, the couple denied intrauterine insemination and selected to proceed with IVF treatment. Ovarian stimulation was carried out with gonadotropin-releasing hormone agonist (GnRHa) down-regulation protocol. On July 22nd, sixteen oocytes were harvested, and fourteen oocytes were fertilized. All embryos were frozen for the prevention of ovarian hyperstimulation syndrome (OHSS) after oocyte retrieval.

In the second menstrual cycle after oocyte retrieval, the patient was admitted to the IVF center for endometrium preparation. Her clinical findings during menstrual period included: the serum hormone levels (estradiol, 42.23 pg/ml, progesterone, 0.22 ng/ml). TVUS revealed thin endometrium of 5 mm and a left ovarian cyst of 25*20 mm. She started with estradiol (E2) valerate (Progynova, Schering, Germany) 3 mg twice daily from day 3 of the cycle onwards. TVUS was used to monitor endometrial development on the 7th and 11th days separately since the artificial cycle was established. Spontaneous ovulation was avoided in the hormone replacement therapy because there was no dominant follicle development under frequent TVUS monitoring. When the endometrial thickness reached 8.2 mm on the 11th day since the artificial treatment, we added vaginal progesterone gel (Crinone 8%, Fleet Laboratories Ltd., United Kingdom) 90 mg daily plus oral dydrogesterone (Dupbaston, Abbott, the Netherlands) 10 mg three times per day. On September 19th, the patient underwent her first FET. Serum β-hCG level was 2.59mIU/ml at fourteenth day following 2 day-3 embryos transfer. Immunochemiluminometric assay was undertaken for testing of serum β-hCG (Cobas8000 e602; Roche Diagnostics GmbH, Mannheim, Germany). The range of detection was between 0.1 and 10000mIU/ml. The sensitivity of the assay was 0.06mIU/ml and the intra-assay coefficient of variation was 10%. Our laboratory is checked for qualification by the External Quality Assessment of Clinical Laboratory Center annually (Ministry of Health of the People’s Republic of China, Beijing, China). Thus, the patient was misdiagnosed as implantation failure and told to stop her luteal phase support. She presented at our clinic during the subsequent vaginal bleeding 5 days later, which was mistaken for menstruation. The clinical findings included: the serum hormone levels (follicle-stimulating hormone, 6.13mIU/ml, luteinizing hormone, 5.63mIU/ml, estradiol, 119.3 pg/ml, progesterone, 0.23 ng/ml); TVUS revealed thin endometrium of 5.7 mm and a left ovarian cyst of 24*15 mm. The patient was suggested using GnRH agonist (Diphereline, 3.75 mg) pretreatment followed by estrogens and progesterone to prepare the endometrium for the next FET cycle. And the re-examination was planned 14 days after GnRHa injection (Table. 1).

**Table 1:** Patient’s clinical presentations, serum hormone trend and timeline of events after frozen embryo transfer

Number of days post-FET	Clinical presentation	Serum β-hCG level (mIU/ml)	Serum estradiol level (pg/ml)	Serum progesterone level (ng/ml)	Event
14	Asymptomatic	2.59	Not measured	Not measured	Stopping luteal support
19	Vaginal bleeding	Not measured	119.3	0.23	GnRHa pretreatment
33	Asymptomatic	4103	1472	> 60	TVUS - GS near posterior uterus wall
34	Asymptomatic	1395	Not measured	Not measured	Day 1 after laparoscopic surgery

*FET* frozen embryo transfer

**Fig. 1:**
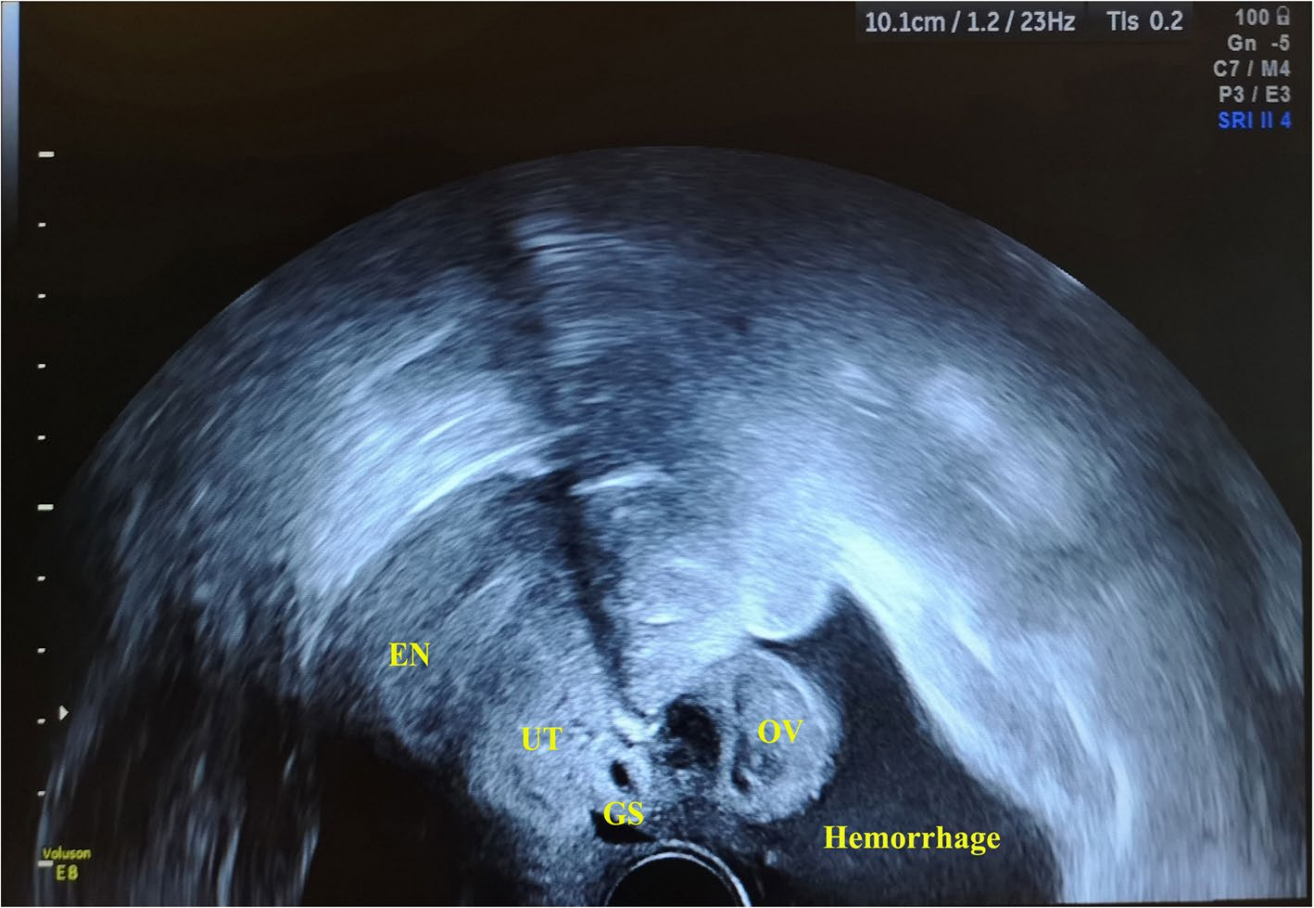
Transvaginal ultrasonography showed the intra-abdominal hemorrhage (96*57 mm) in the pelvic cavity and enlarged ovaries with multiple corpus luteums. It identified a 13 mm × 11 mm-sized GS-like echo near the posterior surface of uterus. EN: endometrium; UT: uterus; GS: gestational sac; OV: ovary

**Fig. 2:**
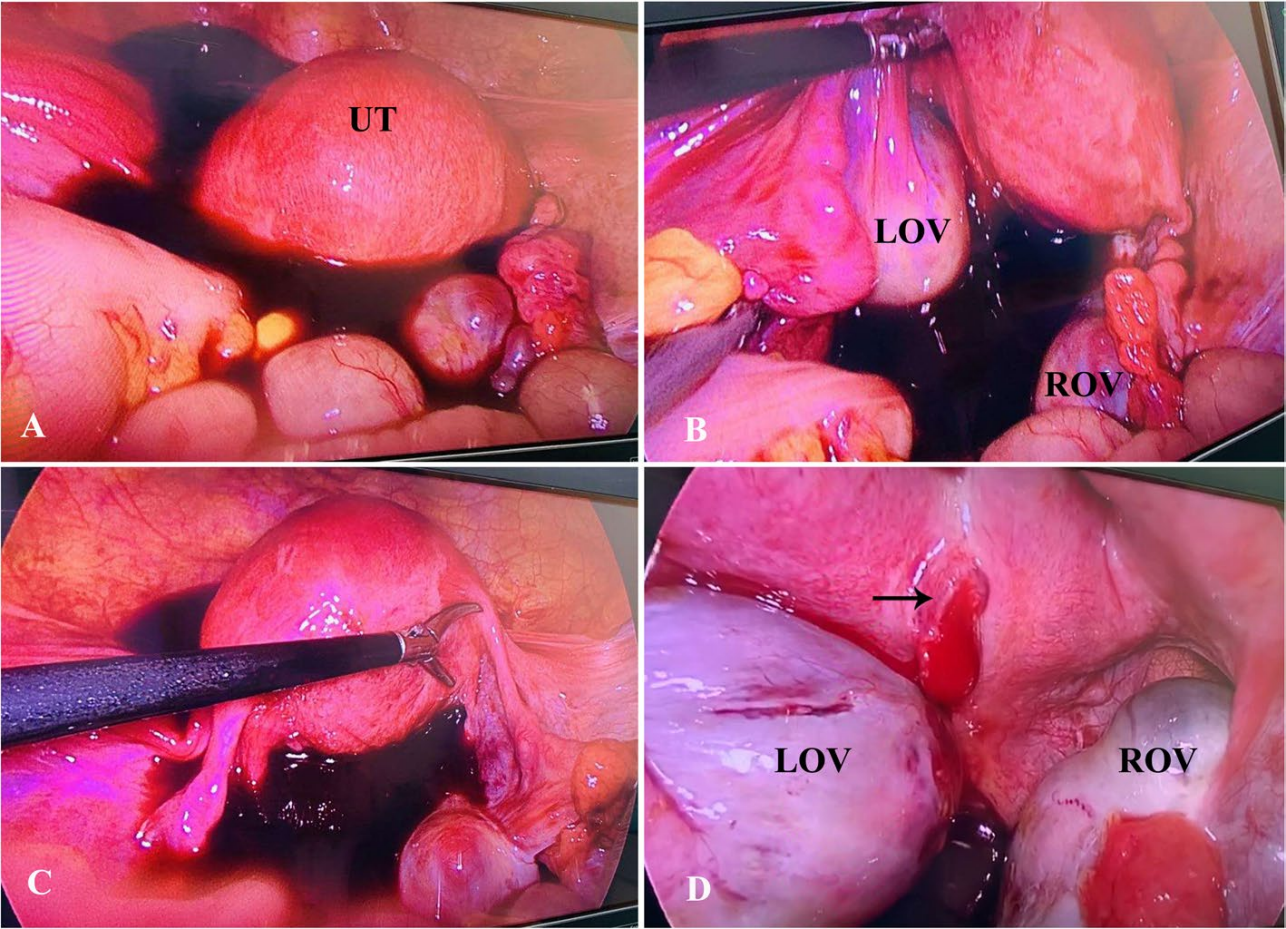
Intraoperative findings during laporascopic surgery: (**A**) Free intraperitoneal hemorrhage with about 400 ml was detected in the pelvis; (**B**) The ovaries were enlarged with multiple cysts, but no ovarian hemorrhage was present. The size of LOV was approximately 65 × 35 mm and the size of ROV was approximately 65 × 39 mm; (**C**) Bilateral fallopian tubes and the shape of uterus appeared normal grossly with no other findings suggesting an EP in the pelvis; (**D**) The ectopic pregnant mass was located on the posterior surface of uterus with active bleeding (black arrow) and its size was approximately 13 × 11 mm. UT: uterus; LOV: left ovary; ROV: right ovary

TVUS was performed 14 days after GnRHa administration (thirty-third day post-embryo transfer) and imaged enlarged ovaries with multiple corpus luteums and the bloody fluid (96*57 mm) in the pelvic cavity. The endometrial thickness was 9 mm and gestational sac (GS)–like echo with a size of 13 × 11 mm was found near the posterior wall of uterus (Fig. 1). The clinical findings included: the serum hormone levels (estradiol, 1472 pg/ml, progesterone, > 60 ng/ml, β-hCG, 4103mIU/ml). The asymptomatic patient was diagnosed with suspected ectopic pregnancy and transported to the department of gynaecology for laparoscopic surgery on the same day. Intraoperatively, abundant hemoperitoneum (400 ml) was found (Fig. 2A). After aspiration of hemoperitoneal fluid, an approximately 10-mm actively bleeding lesion was identified on the posterior surface of uterus where the products of conception were noted (Fig. 2D). Further exploration of the pelvic cavity revealed no signs of uterine perforation and no other findings suggesting an EP anywhere. The tubes appeared grossly normal in appearance (Fig. 2C). Although the ovaries were enlarged with multiple cysts, no ovarian hemorrhage was present (Fig. 2B). Surgeons performed excision of ectopic conception tissue. The operative findings met the Studdiford’s criteria for a primary AEP. The laparoscopic diagnosis was a ruptured abdominal pregnancy. The serum β-hCG level decreased rapidly to 1395mIU/ml on postoperative day 1. Additionally, the final histopathological report confirmed the degenerating chorionic villi and edema of the villous stroma, which combined with blood clots and fibrosis in the tissue removed from the uterine wall.

## Discussion and conclusion

Herein, we reported a primary abdominal pregnancy on the posterior surface of uterus with massive hemopritoneum at early stage of gestation. In previous studies, cases of massive hemoperitoneum associated with abdominal pregnancy before 7 weeks of gestation were rare, which were reported in the omentum, the uterosacral ligament, and the vesicouterine peritoneum [6–8]. These cases suggest that the implantation site may affect the onset of bleeding. In our case, this high-risk patient did not develop any signs of acute abdominal pain and the small abdominal gestational sac can be easily misdiagnosed as hemorrhagic corpus luteum cysts in the setting of abundant hemoperitoneum. Our experience shows that massive intraperitoneal hemorrhage with no gestational sac in the uterine cavity at early gestational stage may be sign of a possible abdominal pregnancy. Thus, these symptoms provide a clue to the early diagnosis of an abdominal pregnancy in early gestation. The clinicians should bear in mind the possibility of this kind of rare condition.

Biochemical pregnancy loss was usually defined as serum β-hCG level > 5mIU/ml 14 days after transferring embryo, and declined to <5mIU/ml at the end without visible gestational sac by ultrasound [9]. In this case, the patient was initially diagnosed as implantation failure 14 days post-FET. We thought the β-hCG concentration as low as 2.59mIU/ml was inappropriate for maintaining gestation. As a result, we did not recheck the serum β-hCG level in the subsequent vaginal bleeding after stopping the luteal phase support. The subsequent vaginal bleeding after stopping luteal support was mistaken as menstruation. Further, the endometrium preparation protocol with GnRHa pretreatment for the next FET was suggested for the patient. As reported, a poorly implanted or dying ectopic pregnancy may be present, in consequence of which the pregnancy test may be negative [10]. Another rare case presented with a very low serum β-HCG level of 3.4 IU/L was also reported to develop an AEP after hormone replacement frozen-thawed cycle [11]. A woman was also reported having undetectable serum β-hCG 9 days after blastocyst transfer, and was then diagnosed with a ruptured AEP and intra-abdominal bleeding 19 days later [12]. Four physiopathologic mechanisms may account for the extreme low or negative serum β-hCG values in ectopic pregnancy: trophoblast degeneration with cessation of hormone production; a very small mass of villi producing the hormone; defective biosynthesis of β-hCG hormone production; enhanced circulatory clearance of the hormone [13]. In the present case, the possibility of a spontaneous pregnancy developed during hormone treatment is quite low because there wasn’t any dominant follicle development under frequent ultrasound monitoring during the period of time in the endometrium preparation within 11 days. As reported, GnRHa causes a flare-up effect during the first week after administration [14]. Therefore, the most plausible explanation for the development of clinical pregnancy in our case is the flare-up of the gonadotropin concentration shortly after pretreatment with GnRHa 3.75 mg injection may rescue the impaired post-implantation development.

Making an early diagnosis of early stage of AEP is so challenging that it is usually missed out or misdiagnosed due to the non-specific signs and symptoms [8]. The mean gestational age at the time of treatment of AEP is reportedly 10 weeks [15]. In the present case, we report a rare case of ruptured primary AEP diagnosed at thirty-third day post-FET, which equals to the gestational age of 7 weeks. The high-resolution ultrasonography plays an important role in diagnosis of AEP and laparoscopy is preferred for hemodynamically stable patients. Our experience shows that AEP could cause serious intra-abdominal bleeding even at earlier stages of pregnancy. What’s more, abundant intraperitoneal hemorrhage and low serum β-hCG levels in early gestation are signs of a possible abdominal pregnancy.

In conclusion, we described a case of abdominal pregnancy following a frozen embryo transfer cycle and summarized our diagnostic experience of this rare type of ectopic pregnancy at early gestational stage. The case highlighted the delayed serum β-hCG rise and massive intraperitoneal hemorrhage may be clues to make early diagnosis of AEP. Clinicians must attach great importance to close monitoring and bear in mind the possibility of abdominal pregnancy.

## Data Availability

Data sharing is not applicable to this article as no datasets were generated or analysed during the current study.

## Abbreviations

AEP: Abdominal ectopic pregnancy
IVF: In-vitro fertilization
FET: Frozen-thawed embryo transfer
hCG: Human chorionic gonadotropin
OHSS: Ovarian hyperstimulation syndrome
TVUS: Transvaginal ultrasound
HSG: Hysterosalpingography
GnRHa: Gonadotropin-releasing hormone agonist
GS: Gestational sac

## References

[1] Rohilla M, Joshi B, Jain V (2018). Neetimala, Gainder S. advanced abdominal pregnancy: a search for consensus. Review of literature along with case report. Arch Gynecol Obstet.

[2] Barel O, Suday RR, Stanleigh J, Pansky M (2019). Laparoscopic removal of an abdominal pregnancy in the pelvic sidewall. J Minim Invasive Gynecol.

[3] Yoder N, Tal R, Martin JR (2016). Abdominal ectopic pregnancy after in vitro fertilization and single embryo transfer: a case report and systematic review. Reprod Biol Endocrinol.

[4] Atrash HK, Friede A, Hogue CJ (1987). Abdominal pregnancy in the United States: frequency and maternal mortality. Obstet Gynecol.

[5] Studdiford WE (1942). Primary peritoneal pregnancy. Am J Obstet Gynecol.

[6] Tanase Y, Yoshida S, Furukawa N, Kobayashi H (2013). Successful laparoscopic management of a primary omental pregnancy: case report and review of literature. Asian J Endosc Surg.

[7] Gundabattula SR, Pochiraju M (2014). Primary abdominal pregnancy in the Uterosacral ligament with Haemoperitoneum: a near miss. J Clin Diagn Res.

[8] Miyauchi A, Yamada M, Furuya M, Matsumura S, Murayama S, Yoshimura Y (2015). Peritoneal pregnancy with massive hemoperitoneum in early gestation: two case reports. Clin Case Rep.

[9] Wu Y, Liu H (2021). Likelihood of live birth with extremely low β-hCG level 14 days after fresh embryo transfer. Gynecol Endocrinol.

[10] Fejgin M, Cohen I, Ben-Nun I, Siegal A, Ben-Aderet N (1986). Acute rupture of an ovarian pregnancy associated with a negative serum B-HCG. Int J Gynaecol Obstet.

[11] Yanaihara A, Ohgi S, Motomura K, Hagiwara Y, Mogami T, Saito K (2017). An abdominal ectopic pregnancy following a frozen-thawed ART cycle: a case report and review of the literature. BMC pregnancy and childbirth.

[12] Irani M, Elias RT, Pereira N, Gunnala V, Rosenwaks Z (2016). Abdominal ectopic pregnancy with undetectable serum β-human chorionic gonadotropin 9 days following blastocyst transfer. J Obstet Gynaecol Res.

[13] Taylor RN, Padula C, Goldsmith PC (1988). Pitfall in the diagnosis of ectopic pregnancy: immunocytochemical evaluation in a patient with false-negative serum beta-hCG levels. Obstet Gynecol.

[14] Wolff MV, Kammerer U, Kollmann Z, Santi A, Dietl J, Frambach T (2011). Combination of gonadotropin-releasing hormone (GnRH) agonists with GnRH antagonists before chemotherapy reduce but does not completely prevent a follicle-stimulating hormone flare-up. Fertil Steril.

[15] Poole A, Haas D, Magann EF (2012). Early abdominal ectopic pregnancies: a systematic review of the literature. Gynecol Obstet Investig.

